# Vaccinia Virus Proteins A52 and B14 Share a Bcl-2–Like Fold but Have Evolved to Inhibit NF-κB rather than Apoptosis

**DOI:** 10.1371/journal.ppat.1000128

**Published:** 2008-08-15

**Authors:** Stephen C. Graham, Mohammad W. Bahar, Samantha Cooray, Ron A.-J. Chen, Daniel M. Whalen, Nicola G. A. Abrescia, David Alderton, Raymond J. Owens, David I. Stuart, Geoffrey L. Smith, Jonathan M. Grimes

**Affiliations:** 1 The Division of Structural Biology and the Oxford Protein Production Facility, Wellcome Trust Centre for Human Genetics, University of Oxford, Oxford, United Kingdom; 2 Department of Virology, Faculty of Medicine, Imperial College London, St. Mary's Campus, London, United Kingdom; University of Alberta, Canada

## Abstract

Vaccinia virus (VACV), the prototype poxvirus, encodes numerous proteins that modulate the host response to infection. Two such proteins, B14 and A52, act inside infected cells to inhibit activation of NF-κB, thereby blocking the production of pro-inflammatory cytokines. We have solved the crystal structures of A52 and B14 at 1.9 Å and 2.7 Å resolution, respectively. Strikingly, both these proteins adopt a Bcl-2–like fold despite sharing no significant sequence similarity with other viral or cellular Bcl-2–like proteins. Unlike cellular and viral Bcl-2–like proteins described previously, A52 and B14 lack a surface groove for binding BH3 peptides from pro-apoptotic Bcl-2–like proteins and they do not modulate apoptosis. Structure-based phylogenetic analysis of 32 cellular and viral Bcl-2–like protein structures reveals that A52 and B14 are more closely related to each other and to VACV N1 and myxoma virus M11 than they are to other viral or cellular Bcl-2–like proteins. This suggests that a progenitor poxvirus acquired a gene encoding a Bcl-2–like protein and, over the course of evolution, gene duplication events have allowed the virus to exploit this Bcl-2 scaffold for interfering with distinct host signalling pathways.

## Author Summary

Cells possess formidable defences against virus infection, but viruses have evolved sophisticated counter-measures to evade such defences. Vaccinia virus, the vaccine used to eradicate smallpox, has about 200 genes, and many of these encode proteins that help the virus evade the host's immune defences. This paper concerns the vaccinia virus proteins A52 and B14, which block signalling pathways leading to the activation of the NF-κB transcription factor and thereby diminish the host immune response to infection. By solving the three-dimensional structures of A52 and B14, we show that they closely resemble a family of cellular and viral proteins (the Bcl-2 family) that usually function to regulate apoptosis (a process by which cells commit suicide, thereby stopping the replication of any viruses with which they are infected). However, neither A52 nor B14 regulate apoptosis. By comparing three-dimensional structures, we show that vaccinia virus Bcl-2–like proteins more closely resemble each other than they do other cellular or viral Bcl-2–like proteins. This suggests that an ancestor of vaccinia virus acquired a gene encoding a Bcl-2–like protein from its host and, over time, this gene has been copied and adapted for different functions within the virus.

## Introduction

*Vaccinia virus* (VACV), the smallpox vaccine, is the prototypic member of the *Poxviridae*; a family of large, double-stranded DNA viruses that replicate in the cytoplasm of host cells [1]. VACV strain Copenhagen was the first poxvirus to be sequenced and encodes about 200 genes [2]. The central portion of orthopoxvirus genomes (∼100 kb) is highly conserved and contains genes essential for virus replication [3]. Genes located towards the termini are more variable and, although non-essential for virus replication, they affect VACV virulence, host range and modulation of the host immune system. These VACV immunomodulators can act either inside or outside infected cells [4]. Intracellular immunomodulators modulate apoptosis, the anti-viral activity of interferons, innate immune signalling and host gene transcription, whilst extracellular immunomodulators inhibit the action of complement, interferons, cytokines and chemokines [4]–[6].

Nuclear factor-κB (NF-κB) is a transcriptional complex that plays a central role in stimulating innate and adaptive immune responses to infection. Receptors for the pro-inflammatory cytokines interleukin (IL)-1 and tumour necrosis factor alpha (TNFα) activate signalling pathways leading to NF-κB activation [7],[8], as do Toll-like receptors (TLRs), which recognise pathogen-associated molecular patterns such as in viral (glyco)proteins and nucleic acids [9],[10]. NF-κB activation downstream of the IL-1 receptor and TLRs requires TNF-receptor-associated factor 6 (TRAF6) and IL-1 receptor associated kinases (IRAKs), while activation downstream of the TNF receptor requires TRAF2 [7],[10]. These independent downstream signalling pathways converge at the IκB kinase (IKK) complex, a key regulator of signalling to NF-κB activation [7]. The importance of the immune response initiated by proteins under NF-κB transcriptional regulation upon virus infection is underscored by the fact that VACV encodes several proteins, A52, A46, B14, K1, N1 and M2, which interfere with the intracellular signalling pathways that lead to the activation of NF-κB [11]–[16].

*A52R* is an immediate-early VACV gene [17] encoding a 23-kDa intracellular protein (A52) that contributes to virus virulence [16]. A52 functions by inhibiting NF-κB activation [14] downstream of the IL-1 receptor and TLRs [18] via interactions with IRAK2 and TRAF6 [16],[18]. While the precise molecular details of these interactions are unclear, the N-terminal death domain of IRAK2 is essential for binding A52 [16], and a truncation mutant of A52 lacking the C-terminal 46 residues (A52_ΔC46_) retains the ability to both bind IRAK2 and inhibit NF-κB activation [18],[19]. Further, a peptide derived from A52 termed ‘P13’, comprising residues 125–135 of A52 plus a nine-arginine cell-transducing sequence (which promotes cellular internalisation of the peptide), inhibits TLR-mediated activation of NF-κB and shows promise as a potent anti-inflammatory therapeutic [20]. In addition to inhibiting NF-κB, A52 activates p38 MAP kinase and enhances the TLR4-induced production of IL-10, a cytokine that inhibits inflammatory and cell-mediated immune responses. Activation of p38 MAP kinase is mediated by the direct binding of A52 to the TRAF domain of TRAF6, and removal of the A52 C-terminal 46 residues abolishes the interaction with TRAF6 and subsequent activation of p38 MAP kinase [16],[19].

VACV strain Western Reserve gene *B14R* is an immediate-early gene [17],[21] that encodes a 17-kDa cytosolic protein (B14) that contributes to VACV virulence [21] and inhibits the IKK complex [13]. The interaction of B14 with the IKK complex depends on the presence of IKKβ, and B14 bound to the IKK complex prevents phosphorylation of the IKKβ activation loop. Consequently, B14 inhibits the phosphorylation and subsequent ubiquitin-mediated degradation of IκBα, the inhibitor of NF-κB [13]. In this way, B14 blocks activation of NF-κB downstream of a variety of stimuli including TNFα, IL-1, poly(I∶C) and phorbol myristate acetate [13].

While B14 and A52 share identifiable sequence similarity with each other, being members of a Pfam [22] protein family that also includes VACV proteins A46, K7, C6 and C16/B22, they do not display significant sequence similarity to other cellular or viral proteins [14],[15],[21],[23]. Protein structure is more strongly conserved during evolution than protein sequence [24] and determination of virus protein structures is a powerful tool for identifying previously undetermined functional and evolutionary relationships [25]. For example, the recent crystal structures of VACV N1 [26],[27] and myxoma virus M11 [28],[29] revealed that these proteins possess Bcl-2–like structures despite sharing no identifiable sequence similarity with the cellular Bcl-2 family of proteins. Members of the Bcl-2 family are small α-helical proteins that can be either pro- or anti-apoptotic and they regulate the release of pro-apoptotic molecules from mitochondria [30]. The structures of N1 and M11 both display conserved features important for anti-apoptotic Bcl-2 function; namely the presence of an elongated surface groove for binding α-helical motifs (BH3 peptides) of pro-apoptotic Bcl-2–like proteins and thereby antagonizing their function. Functional studies performed in light of these structure elucidations confirmed that N1 and M11 protect cells from apoptosis by binding to pro-apoptotic Bcl-2 family members such as Bid and Bax [26],[29], the affinity [27],[28] and binding mode [29] being comparable to cellular anti-apoptotic Bcl-2 family proteins. The structural and functional similarity of the poxvirus and cellular Bcl-2–like proteins suggest that these viral proteins had a cellular origin, a common theme in poxviruses and other large DNA viruses [31]–[33].

To understand the mechanism of action and evolutionary origins of VACV A52 and B14 in more detail, we have solved their crystal structures at 1.9 Å and 2.7 Å resolution, respectively. We show that both proteins are Bcl-2 family members but lack a surface groove for binding BH3 peptides and, consonant with this, are not anti-apoptotic. Instead these proteins have acquired the ability to inhibit signalling pathways that activate NF-κB.

## Results/Discussion

**Figure 1:**
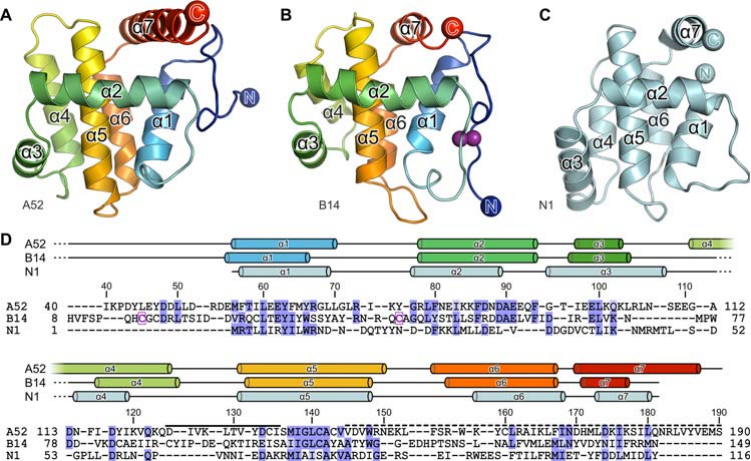
Vaccinia virus (VACV) proteins A52 and B14 share a Bcl-2–like fold. (A) A52 and (B) B14 are shown as ribbons, ramp coloured from blue (N terminus) to red (C terminus). The disulphide bond observed in B14 is shown as purple spheres. (C) The structure of the poxvirus Bcl-2–like protein N1 (cyan; PDB ID 2uxe). (D) Structure-based sequence alignment of the VACV Bcl-2–like proteins. Residues that are highly or moderately conserved (BLOSUM62 score) are coloured marine and light blue, respectively, and cysteine residues that form the disulphide bond observed in B14 are boxed and in purple face. Residues of A52 that encompass the ‘P13’ peptide and residues removed in the A52_ΔC46_ mutant, which does not bind TRAF6, are marked with a solid and a dashed line, respectively. The secondary structures of A52, B14 and N1 are shown above the sequences with α helices represented as cylinders.

The structures of A52 and B14 were solved by multiple-wavelength anomalous dispersion (MAD) experiments performed upon crystals of selenomethionine (SeMet)-labelled protein (Tables 1 & 2). The structures of two independent crystal forms of an N-terminal truncated form of A52 comprising residues 37–190 (A52_ΔN36_) were refined to 1.9 Å and 2.8 Å resolution with residuals R/R_free_ = 0.177/0.215 and 0.181/0.192, respectively, with residues 40–189 being observed in electron density maps in both crystal forms (Figure 1A). Residue 190 and the first two residues of the polyHis purification tag were also visible in the low-resolution structure. Both crystal forms of A52 contain 2 molecules per asymmetric unit arranged as a dimer, the overall fold (0.32±0.07 Å root-mean-squared displacement [rmsd] over 150 C^α^ atoms) and dimer interface being highly conserved (Figure S1). The structure of SeMet B14 was refined to 2.7 Å resolution with residuals R/R_free_ = 0.223/0.244, residues 8–149 being observed in the electron density (Figure 1B). Four molecules of B14 are present in the crystallographic asymmetric unit, forming two dimers with highly conserved overall folds (0.11±0.02 Å rmsd over 142 C^α^ atoms) and dimer interfaces (Figure S1).

**Table 1:** Data collection statistics.

	Unlabelled A52		SeMet A52			SeMet B14		
	*High resolution*	*Low resolution*	*Peak*	*Inflection*	*Remote*	*Peak*	*Inflection*	*Remote*
Beamline	ESRF ID14-4	Diamond I03	ESRF BM14			ESRF BM14		
Wavelength (Å)	0.9395	1.060	0.9788	0.9790	0.8856	0.9789	0.9792	0.8856
Resolution limits (Å) a	50–1.9 (1.93–1.90)	50.0–2.8 (2.80–2.75)	24.9–2.1 (2.20–2.09)	30.9–2.3 (2.42–2.29)	35.6–2.3 (2.42–2.29)	50.0–2.9 (3.00–2.90)	50.0–2.7 (2.82–2.72)	50.0–2.7 (2.80–2.70)
Space group	*P*2_1_	*R*3∶H		*P*2_1_			*C*2	
Unit cell dimensions (Å, °)	46.2, 59.1, 75.7, 106.6	126.1, 126.1, 124.0		46.4, 59.2, 75.5, 106.5			168.7, 43.9, 99.9, 112.1	
Unique reflections a	30,971 (1,544)	19,184 (972)	23,415 (3,318)	17,762 (2,544)	17,783 (2,548)	14,859 (1,446)	18,657 (1,798)	19,127 (1,878)
Redundancy a	4.1 (4.1)	5.7 (4.9)	7.6 (7.5)	3.8 (3.8)	3.8 (3.8)	6.9 (6.5)	3.4 (3.2)	3.7 (3.4)
Completeness (%) a	99.9 (99.7)	100.0 (100.0)	99.4 (96.5)	99.6 (98.1)	99.6 (97.9)	99.2 (99.7)	99.5 (97.8)	99.8 (98.6)
<*I*/σ(*I*)> a	12.1 (1.9)	13.8 (2.1)	11.5 (3.0)	9.3 (2.6)	7.1 (1.8)	23.7 (5.5)	11.3 (1.6)	10.4 (1.8)
*R*_merge_ a, b	0.080 (0.714)	0.093 (0.815)	0.136 (0.611)	0.112 (0.426)	0.163 (0.617)	0.104 (0.398)	0.096 (0.654)	0.124 (0.839)

a Numbers in parentheses are for the highest resolution shell.

b *R*_merge_ = Σ_hkl_Σ_i_|*I(hkl;i)*−<*I(hkl)*>|/Σ_hkl_Σ_i_ *I(hkl;i)*, where *I(hkl;i)* is the intensity of an individual measurement of a reflection and <*I(hkl)*> is the average intensity of that reflection.

**Table 2:** Refinement statistics.

	High resolution A52	Low resolution A52	B14
Space group	*P*2_1_	*R*3∶H	*C*2
Resolution limits (Å) a	33.8–1.9 (1.95–1.90)	40.1–2.8 (2.89–2.75)	46.3–2.7 (2.76–2.70)
Reflections in working set a	29,385 (2,161)	19,183 (2,611)	36,439 (2,577)
Reflections in test set a	1,571 (86)	988 (135)	1,783 (99)
*R* factor of working set a, b	0.177 (0.252)	0.181 (0.287)	0.223 (0.381)
*R*_free_ (%) a, b, c	0.215 (0.298)	0.192 (0.302)	0.244 (0.412)
Number of atoms (protein/water)	2,508/207	2,516/0	4,632/0
Number of atoms with alternate conformations (protein/water)	36/0	12/–	0/–
Residues in Ramachandran favoured region (%)	99.3	98.0	97.7
Ramachandran outliers (%)	0.0	0.0	1.4
rmsd bond lengths (Å)	0.010	0.005	0.005
rmsd bond angles (°)	1.107	0.816	0.777
Average *B* factors (Å^2^) (protein/water)	29.6/38.0	81.7/–	62.5/–

a Numbers in parentheses are for the highest resolution shell.

b *R* = Σ_hkl_∥*F*_obs_ *(hkl)*|−|*F*_calc_ *(hkl)*∥/Σ_hkl_ |*F*_obs_ *(hkl)*|, where |*F*_obs_ *(hkl)*| and |*F*_calc_ *(hkl)*| are the observed and calculated structure facture amplitudes.

c *R*_free_ equals the *R*-factor of test set (5% of the data removed prior to refinement).

**Figure 2:**
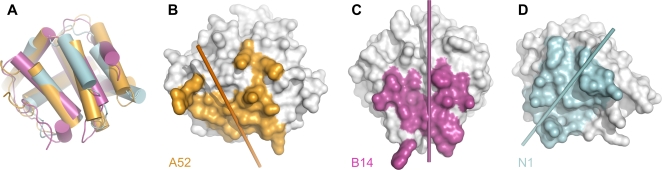
A52, B14 and N1 utilise a conserved face for dimerisation, but the 2-fold rotations that relate monomers of the dimers differ significantly. (A) Superposition of A52 (orange), B14 (magenta) and N1 (cyan; PDB ID 2uxe). (B–D) The molecular surfaces of (B) A52, (C) B14 and (D) N1 are shown in white, residues that form intermolecular (dimer) contacts being coloured orange (A52), magenta (B14) or cyan (N1). The molecules are oriented as in (A). Cylinders represent the two-fold rotation axes of the respective dimers.

The structures of A52 and B14 are entirely α helical, both comprising seven α helices and adopting the Bcl-2–like fold (Figure 1). The Bcl-2–like folds of A52 and B14 are surprising given the low sequence similarity shared between A52, B14 and other Bcl-2–like proteins (Table 3), and the structures contradict a previous suggestion that A52 would adopt a fold like Toll-like–IL-1 resistance (TIR) domains [14]. A structural similarity search using SSM [34] identified the closest relative of both A52 and B14 to be N1 (Figure 1C), a VACV Bcl-2–like protein that inhibits apoptosis [26],[27] and which has also been reported to inhibit signalling pathways leading to activation of NF-κB [35]. N1, A52 and B14 all homodimerise both *in crystallo* and *in vitro* (Figure S1, [36] and data not shown). While the face of the structure involved in dimerisation is generally conserved across these proteins, the orientations of the two-fold rotation axes that relate the monomers differ by up to 57° (Figure 2). Despite being a cytosolic protein, and despite being purified and crystallised in the presence of the reducing agent β-mercaptoethanol, a single disulphide bond is observed in B14 between cysteine residues 15 and 46, locking the N-terminal loop with the α1-α2 loop (Figure 1B). There is no obvious functional role for this disulphide bond other than stabilising the structure of the protein.

**Table 3:** Structural and sequence similarity of poxvirus, herpesvirus and cellular Bcl-2–like proteins.

	VACV A52 (2vvw)	VACV B14 (2vvy)	VACV N1 (2i39)	Myxoma virus M11 (2jbx)	Murine gamma-herpesvirus 68 Bcl-2 like (3bl2)	Mouse Bcl-x_L_ (2bzw)	Human Bid (2bid)
VACV A52 (2vvw)	–	1.91 (124)	1.96 (108)	2.46 (107)	2.49 (100)	2.72 (111)	2.84 (91)
VACV B14 (2vvy)	21.8	–	2.33 (101)	2.46 (95)	2.55 (105)	2.79 (103)	2.51 (89)
VACV N1 (2i39)	18.5	15.8	–	2.29 (101)	2.36 (97)	2.75 (103)	2.83 (84)
Myxoma virus M11 (2jbx)	11.2	5.3	9.9	–	2.38 (105)	2.33 (109)	2.90 (83)
Murine gamma-herpesvirus 68 Bcl-2 like (3bl2)	5.0	11.4	14.4	5.7	–	2.26 (112)	3.13 (95)
Mouse Bcl-x_L_ (2bzw)	3.6	8.7	8.7	11.0	20.5	–	2.84 (105)
Human Bid (2bid)	6.6	6.7	13.1	9.6	10.5	10.5	–

PDB IDs for the structures used in the comparisons are shown in parentheses. C^α^ rmsds and numbers of equivalent residues (in parentheses) are shown above the diagonal, sequence identities of equivalent residues (%) are shown below.

**Figure 3:**
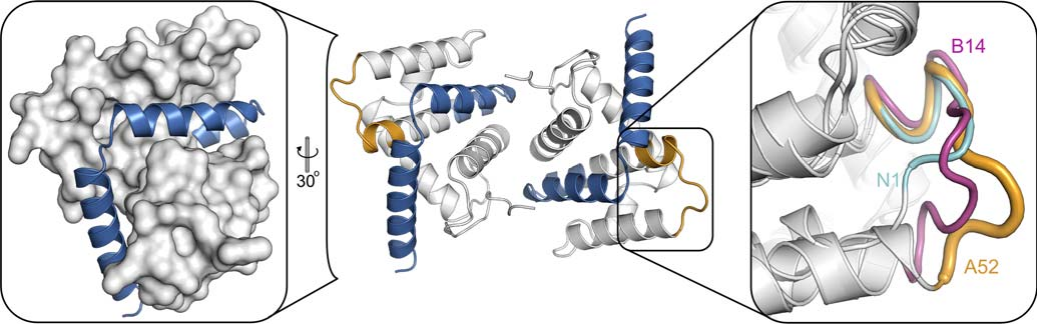
Regions of A52 implicated in TRAF6 and IRAK2 interactions. The A52 dimer is shown in ribbon representation with residues that encompass the ‘P13’ peptide (residues 125–135) coloured orange and residues removed in the A52_ΔC46_ mutant (residues 145–189) coloured blue. While the putative IRAK2-interacting loop is exposed on the surface of A52, residues 145–189 form part of the core of the protein. (Left inset) The molecular surface of A52_ΔC46_ is shown in white, with A52 residues removed in this mutant (residues 145–189) shown as a blue ribbon. (Right inset) The structures of A52, B14 and N1 are shown superposed. Thicker coloured tubes denote the regions equivalent to residues 125–135, encompassing the ‘P13’ peptide, of A52 (orange): B14 residues 89–102 (magenta) and N1 residues 63–70 (cyan).

Residues 125–135 of A52, which encompass the 11-residue ‘P13’ peptide sequence that in isolation can inhibit NF-κB activation [20], form the surface loop between helices 4 and 5. This is distal to the A52 dimerisation interface and is appreciably different in length, amino acid composition and conformation to the comparable loops in the other VACV Bcl-2–like proteins (Figures 1 & 3). It is a likely candidate for mediating the interaction with IRAK2, although such identification awaits direct experimental confirmation. The final 46 residues, removed in the A52_ΔC46_ truncation mutant that abolished TRAF6 binding and the ability to stimulate p38 MAP kinase, comprise the final 6 residues of helix 5 and all of helices 6 and 7. Given that helices 5 and 6 contribute to the central core of the protein, it is unclear whether this truncated protein would be stable and natively folded (Figure 3) let alone whether it would dimerise like the native protein (900 Å^2^ of the 1,300 Å^2^ of surface area buried by the native dimer interface being abolished by the truncation). Further study is therefore required to identify the binding face of TRAF6 on the surface of A52.

**Figure 4:**
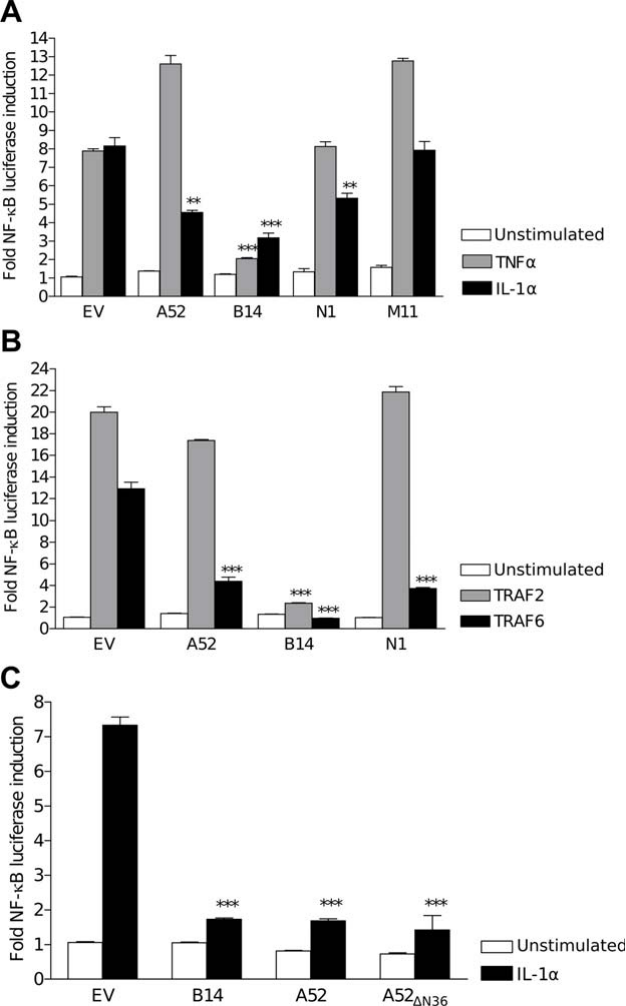
The relative effects of A52, B14, N1 and M11 on signalling to NF-κB. (A) VACV Bcl-2–like proteins affect signalling to NF-κB downstream of IL-1α and TNFα differently. HEK 293 cells were transfected with FLAG-tagged expression alleles for A52, B14, N1, M11 or empty vector (pCI; EV) together with an NF-κB reporter plasmid and *Renilla* internal control. Cells were treated with 100 ng/ml of IL-1α, TNFα or left untreated for 8 h, lysed and assayed for NF-κB–inducible luciferase activity. (B) B14, but not A52, blocks activation of NF-κB downstream of TRAF2 and TRAF6. HEK 293 cells were transfected with FLAG-tagged expression alleles for A52, B14, N1, M11 or empty vector (pCI; EV) together with plasmids for NF-κB reporter gene, *Renilla* internal control and either TRAF2 or TRAF6. Cells were lysed 24 h post-transfection and measured for NF-κB–inducible luciferase activity. (C) The N terminus of A52 is not required for inhibition of NF-κB downstream of IL-1α. HEK 293 cells were transfected with pOPINE vectors containing C-terminally His-tagged full length A52 or C-terminally His-tagged A52 lacking residues 1–36 (A52_ΔN36_), FLAG-tagged B14 or empty vector (pOPINE; EV) together with NF-κB reporter and *Renilla* internal control. Cells were treated as in (A). Data are expressed as means±standard deviation of 2–4 independent experiments. Statistics: two-tailed Student's t-Test (*P<0.05, **P<0.005 ***P<0.0005).

The determination of the structures of VACV proteins B14 and A52 establishes that poxviruses encode a family of Bcl-2–like proteins that also includes VACV N1 and myxoma virus M11. These proteins have been reported to have diverse functions including inhibition of apoptosis and interference with signalling from several stimuli leading to activation of NF-κB. We therefore undertook a direct comparison of the ability of these proteins to inhibit apoptosis in response to staurosporine or inhibit NF-κB activation in response to IL-1α or TNFα stimulation (Figure 4A) or over-expression of TRAF2 or TRAF6 (Figure 4B). A52, B14 and N1 all inhibit the activation of NF-κB to some degree in response to IL-1α or downstream of TRAF6. However, only B14 is able to block signalling downstream of TNFα/TRAF2. This is due to the ability of B14 to inhibit signalling from the IKK complex via its interaction with IKKβ: the nexus where IL-1 receptor, TNF receptor and TLR signalling pathways to NF-κB converge [13]. The ability of A52 to inhibit IL-1α– but not TNFα–induced signalling is consistent with previous findings [14],[16], whereas the inability of N1 to inhibit TNFα signalling is inconsistent with a prior report [35]. The mechanism by which N1 inhibits signalling downstream of IL-1α is unclear. However, given that N1 inhibits TRAF6-induced NF-κB activation but does not interact with the IKK complex [13], contrary to previous suggestions [35], it is likely that it acts at the level of TRAF6 or on downstream proteins in the pathway that precede the IKK complex. M11 did not inhibit either pathway, consistent with a primarily anti-apoptotic function during virus infection [29]. To test whether the N terminus of A52, predicted to be unstructured and removed for crystallisation, is required for anti–NF-κB activity, full length A52 and the A52_ΔN36_ truncation were compared for their ability to block signalling induced by IL-1α (Figure 4C). A52_ΔN36_ had a comparable inhibitory effect to the full-length protein, indicating that the N-terminal 36 residues are not necessary for the inhibitory action of A52.

**Figure 5:**
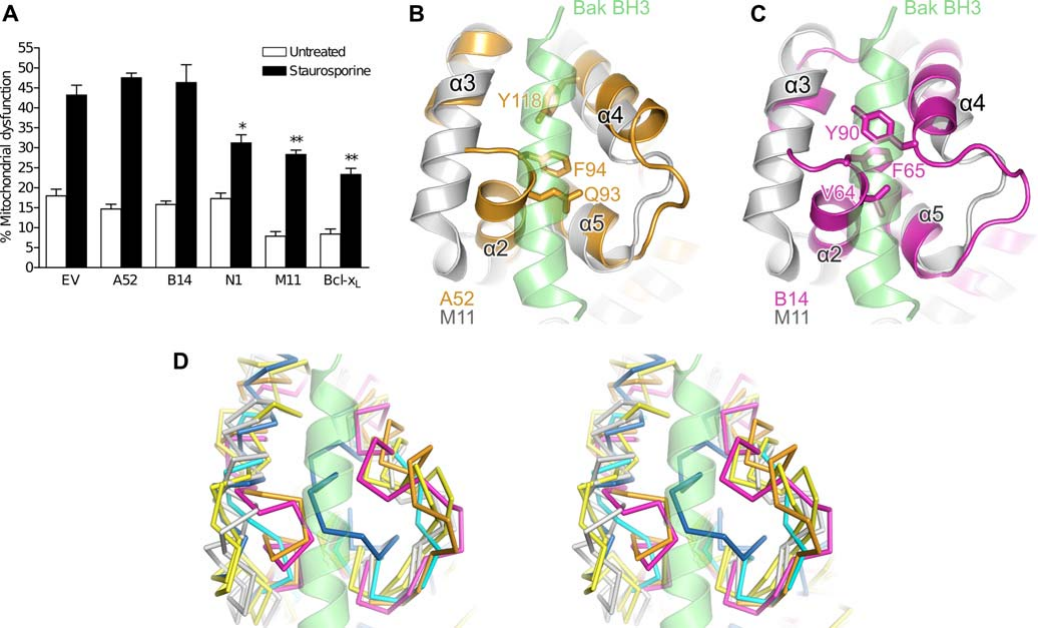
A52 and B14 lack a surface BH3-peptide binding groove and do not block apoptosis. (A) HeLa cells were transfected with vectors expressing FLAG-tagged A52, B14, N1, M11, untagged Bcl-x_L_ or empty control vector (pCI; EV) together with CD20 expression vector. After a further 24 h, cells were treated with 0.5 µM staurosporine for 1 h or left untreated and assayed for mitochondrial dysfunction by JC-1 staining and FACS analysis. Data are expressed as means±standard deviation of 3 independent experiments. Statistics: two-tailed Student's t-Test (*P<0.05, **P<0.005). (B and C) The BH3-peptide binding groove of M11 is occluded in both A52 and B14. The structures of (B) A52 (orange ribbon) and (C) B14 (magenta ribbon) are shown superposed upon the structure of myxoma virus M11 (light grey ribbon) in complex with the BH3 peptide of human Bak-2 (lime green helix; PDB ID 2jby). Side chains that block the BH3-peptide binding groove are shown as sticks. (D) Stereogram depicting superposed C^α^ traces of A52 (orange), B14 (magenta), VACV N1 (cyan; PDB ID 2uxe), myxoma virus M11 (light grey; PDB IDs 2jbx and 2jby), mouse Bcl-x_L_ (yellow; PDB IDs 1pq0 and 1pq1) and Epstein-Barr virus BHRF1 (dark blue; PDB ID 1q59). Two conformations of M11 and Bcl-x_L_ are shown (in the presence and absence of bound BH3 peptide). The BH3 peptide of human Bak-2 in complex with myxoma virus M11 is shown (lime green helix; PDB ID 2jby).

Anti-apoptotic Bcl-2–like proteins inhibit apoptosis by binding the BH3 peptides of pro-apoptotic Bcl-2–like and BH3-only proteins in a hydrophobic groove on the surface of the anti-apoptotic protein [30]. While both N1 and M11 block mitochondrial apoptosis in cells treated with the pan-kinase inhibitor staurosporine (Figures 5A & S2), consistent with previous reports [26],[29], neither B14 nor A52 block apoptosis (Figures 5A & S2). Superposition of our structures onto that of myxoma virus M11 in complex with the BH3 peptide of Bak [29] reveals the hydrophobic BH3-peptide binding grooves are occluded in both A52 and B14, but not in N1 (Figure 5). Residues 92–94 and 117–122 block the groove in A52, the C-terminal ends of helices 2 and 4 being oriented closer to each other than in M11, thereby narrowing the groove significantly, and the side chains of Q93, F94 and Y118 pointing directly into and filling the groove. Equivalent residues (63–66 and 82–90) occlude the groove of B14, helices 2 and 4 again being much closer to each other than in M11 and three side chains (V64, F65 and Y90) pointing directly into and blocking the groove. Bcl- x_L_ and BHRF1, two Bcl-2 family proteins that are able to bind BH3 peptides and thereby inhibit apoptosis, have restricted grooves that are ‘closed’ in the absence of bound BH3 peptide [37]–[39]. Movement of helices 3 and 4 widens the Bcl-x_L_ groove to allow BH3-peptide binding [39] and, while a structure of BHRF1 in complex with a BH3 peptide is not available, it is likely that rearrangement of the loop between helices 4 and 5 would be required for BH3-peptide binding. In contrast, blockage of the BH3-peptide binding groove by residues at the C-terminal end of helix 2 is characteristic of A52 and B14 (Figure 5D) and we propose that it explains the inability of A52 and B14 to protect cells from apoptotic challenge. Interestingly, helix 2 of N1 has the same orientation as in A52 and B14 but is one turn shorter and thus doesn't occlude the BH3-peptide–binding groove (Figure 5D). This is consistent with both the ability of N1 to inhibit apoptosis (Figures 5A and S2) and suggests that N1 is an evolutionary ‘intermediate’ between the apoptosis-regulating Bcl-2–like proteins and those that inhibit NF-κB activation.

**Figure 6:**
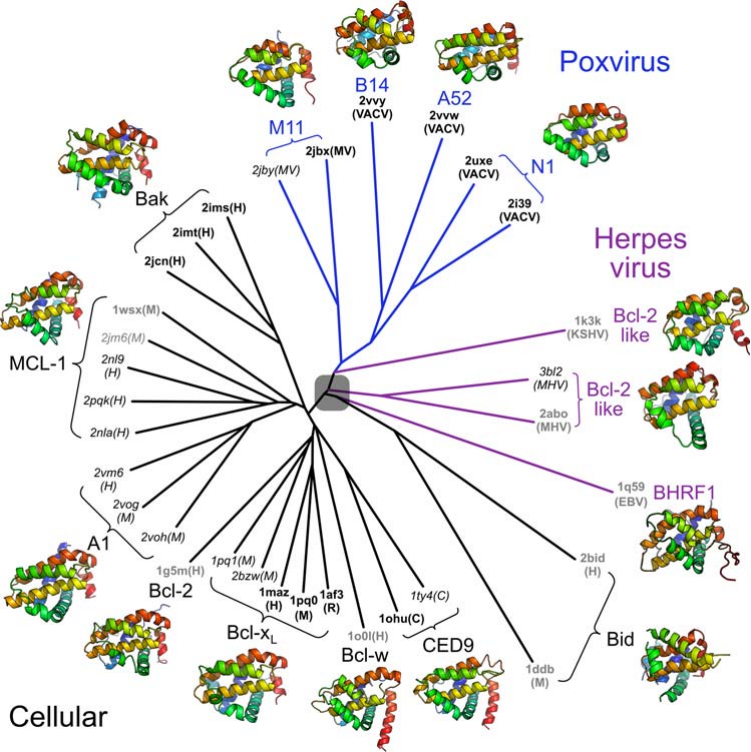
Structure-based phylogenetic analysis of virus and cellular Bcl-2–like proteins. The structures were superposed and a pairwise distance matrix was constructed as described in *Materials and Methods*. PDB codes for each structure used are given followed by their species of origin in parentheses [Human (H), Mouse (M), Rat (R), *C. elegans* (C), Vaccinia virus (VACV), Myxoma virus (MV), Kaposi sarcoma herpes virus (KSHV), murine gamma-herpesvirus 68 (MHV), Epstein-Barr virus (EBV)]. Structures determined by X-ray crystallography are labelled in black, NMR models in grey and structures of Bcl-2–like proteins in complex with BH3 peptides are italicised. It is not certain that all Bcl-2–like proteins share a common ancestor and, as such, the root of the tree is shaded. Ribbon diagrams of representative structures for each protein are colour ramped from blue (N terminus) to red (C terminus).

To investigate the evolutionary relationship between A52, B14, N1 and other Bcl-2–like proteins further, a structure-based phylogenetic tree [25] was calculated using all-pairs pairwise structural superpositions of A52, B14 and 30 representative cellular and viral Bcl-2–like proteins (Figure 6). Amongst the cellular Bcl-2–like proteins, orthologues are seen to cluster together, residing closer to each other on the tree than to paralogue structures from the same species. However, the poxvirus Bcl-2–like proteins are all more similar to each other than they are to cellular Bcl-2–like proteins despite their divergent functions. The closest viral relatives to the cellular Bcl-2–like proteins are the proteins from gamma herpes viruses (EBV, KSHV and MHV-68) and M11. These proteins bind pro-apoptotic BH3-peptides and are potent inhibitors of apoptosis [28],[29],[37],[38],[40],[41]. More distantly related is N1, which both inhibits apoptosis by binding BH3-peptides and interferes with NF-κB activation (Figures 4 & 5). The proteins most distinct from cellular Bcl-2–like proteins, A52 and B14, lack a BH3-peptide binding groove and inhibit NF-κB activation rather than apoptosis (Figure 5). This is consistent with the hypothesis that an ancestral poxvirus acquired a gene encoding a Bcl-2–like protein from its host and over evolution this useful protein scaffold has been adapted to interfere with several different cellular signalling pathways [31]–[33]. As poxvirus and cellular Bcl-2–like proteins are of a similar size, convergence of separately-acquired cellular Bcl-2–like proteins to this common, minimal fold in the virus seems unlikely. Interestingly, the poxvirus Bcl-2–like proteins are more closely related to Bcl-2–like proteins from herpes viruses than they are to cellular Bcl-2–like proteins. This might imply either some elements of common ancestry of poxviruses and herpes viruses, independent acquisition of the same cellular Bcl-2–like protein by both virus families, or horizontal gene transfer between the viruses. While Bid lies closer to viral than cellular Bcl-2–like proteins, it is only distantly related to either group (Tables 3 & S1). Its position in the tree most likely arises from a form of ‘long-branch attraction’ [42] and does not reflect a close evolutionary relationship with viral Bcl-2–like proteins.

We have now shown that three VACV proteins, N1, A52 and B14, all adopt the Bcl-2–like fold despite sharing little sequence identity and having different functions. A52 and B14 are both members of a protein family (PF06225) that also includes VACV proteins A46 (VACWR172), C6 (VACWR022), C16/B22 and K7 (VACWR039) (Figure S3) [14],[23] and it is likely that these proteins will also adopt a Bcl-2–like fold. Like B14 and A52, A46 inhibits activation of NF-κB, although it does so by an independent mechanism, binding directly to the TIR domains of TLR4 and of the TLR-associated proteins MyD88, TRIF, TRAM and Mal [14],[15]. The sequence similarity shared between A52, B14 and A46 make it likely that A46 will share the Bcl-2 fold, rather than the TIR fold as suggested previously [15]. The functions of C6, C16/B22 and K7 are unknown, although peptides derived from C6 and C16/B22 are presented to CD8^+^ cells by MHC-I during VACV infection, confirming that these proteins are expressed [43],[44]. [Note that C16/B22 is distinct from the vaccinia serpin-like serine protease inhibitor SPI-1 (also known as B22)] [45],[46]. Temporal profiling of VACV gene expression during infection shows A52, B14, C6 and K7 to be immediate-early genes and A46 to be an early gene [15],[17],[21],[36]. Genes expressed immediately after or early during infection are often associated with modulation of the host immune response [1],[17]. Therefore, immediately after or early during infection poxviruses express a number of proteins that adopt the Bcl-2–like fold. While the precise roles of C6, K7 and C16/B22 remain to be determined, it is evident that poxviruses have adapted the Bcl-2–like fold to interfere at multiple points along signalling pathways mediating the host immune response to viral infection.

In summary, we have solved the structure of the VACV immunomodulatory proteins A52 and B14. These proteins both adopt the Bcl-2–like fold, despite sharing little sequence identity with viral or cellular Bcl-2–like proteins. Neither A52 nor B14 inhibit apoptosis as they lack a hydrophobic surface groove capable of binding pro-apoptotic BH3 peptides. Further, A52 and B14 are representative members of a protein family that also includes VACV proteins K7, C6, A46 and C16/B22, and it is likely that all these proteins will adopt a Bcl-2–like fold despite their varied functions. Structure-based phylogenetic analysis of viral and cellular Bcl-2–like proteins reveal that poxvirus Bcl-2–like proteins are more similar to each other than they are to cellular Bcl-2–like proteins, implying that these poxvirus Bcl-2–like proteins share a common evolutionary origin.

## Materials and Methods

### Expression plasmids

Full-length A52 and an N-terminal truncation lacking residues 1–36 (A52_ΔN36_) were amplified from VACV Western Reserve (WR) cDNA using KOD HiFi DNA polymerase (Novagen) according to the manufacturer's instructions (forward primer: 5′- AGGAGATATACCATGGACATAAAGATAGATATTAGTATTTCTGG-3′, reverse primer: 5′-GTGATGGTGATGTTTTGACATTTCCACATATACTAGTCTATTC-3′ [A52] and forward primer: 5′-AGGAGATATACCATGACTGATGTTATCAAACCTGATTATCT-3′, reverse primer: 5′-GTGATGGTGATGTTTTGACATTTCCACATATACTAGTCTATTC-3′ [A52_ΔN36_]). The genes were cloned into pOPINE (adding a C-terminal Lys-His_6_ fusion tag) by InFusion cloning [47]. Full-length B14 (residues 1–149) was amplified by PCR from VACV WR cDNA using HiFi *Taq* DNA polymerase (Geneaid) with primers containing NdeI and EcoRI restriction sites respectively (forward primer: 5′- GGAATTCCATATGACGGCCAACTTTAGTACC-3′, reverse primer: 5′-CTCGAATTCTCATCAATTCATACGCCGGAA-3′; sequences encoding restriction sites are underlined). The PCR product was cloned into pET28a (Promega) cut with NdeI and EcoRI (Roche) using T4 DNA ligase (Promega), the resultant plasmid encoding full-length B14 with an N-terminal His_6_ fusion tag.

pCI-based expression plasmids for FLAG-tagged N1 and B14 were constructed as described previously [13],[26]. Plasmids containing FLAG-tagged A52, TRAF2, TRAF6, NF-κB luciferase reporter and pTK-*Renilla* luciferase internal control were gifts from Dr. Andrew Bowie (Trinity College, Dublin, Ireland). FLAG-tagged M11 was a gift from Dr. David Huang (The Walter and Eliza Hall Institute of Medical Research, Melbourne, Australia) and Bcl-x_L_ was provided by Professor Xin Lu (Ludwig Institute for Cancer Research, London, United Kingdom). Relative protein expression levels were verified by immunoblotting using anti-FLAG or anti-Bcl-x_L_ monoclonal antibodies.

### Large-scale expression

Both unlabelled and selenomethionine (SeMet)-labelled A52_ΔN36_ were overexpressed by autoinduction in *E. coli* Rosetta(DE3)pLysS cells [48] and stored frozen (−80°C) until required. The final mass obtained for cells expressing SeMet-labelled A52_ΔN36_ (0.5 g/L) was much lower than obtained for those expressing the unlabelled protein (∼10 g/L). SeMet-labelled B14 was expressed in *E. coli* B834(DE3) cells (grown in the presence of 30 µg/mL kanamycin) using SeMet medium (Molecular Dimensions) supplemented with 40 mg/L l-SeMet. Cultures were grown at 37°C to an optical density (OD_600_) of 0.6, cooled to 20°C and protein expression was induced by the addition of 1 mM isopropyl β-d-thiogalactopyranoside. Cells were harvested after 16 h incubation by centrifugation (5,500 *g*, 4°C, 20 min) and the cell pellet was stored frozen (−80°C) until required.

### Purification of A52_ΔN36_

Thawed cells were resuspended in 50 mM Tris pH 7.5, 500 mM NaCl, 20 mM imidazole, 0.2% v/v TWEEN-20, supplemented with ∼5 mg hen egg-white lysozyme (Sigma) and 1 EDTA-free protease inhibitor tablet (Roche), and incubated for 1 h at 20°C before being sonicated on ice for 5 min until the cells were lysed completely. The cell lysate was cleared by centrifugation (45,000 *g*, 8°C, 30 min) and incubated with 1–2 mL of Ni Sepharose 6 Fast Flow beads (GE Healthcare) for 1 h at 4°C before being loaded into a disposable chromatography column (BioRad). Beads were washed with 15 c.v. of 50 mM Tris pH 7.5, 500 mM NaCl, 20–40 mM imidazole and the protein was eluted in 50 mM Tris pH 7.5, 500 mM NaCl, 500 mM imidazole. Pooled fractions containing A52_ΔN36_ were diluted 10-fold in gel-filtration buffer (20 mM Tris-HCl pH 7.5, 200 mM NaCl), concentrated and then applied to a Superdex 75 column (GE Healthcare) equilibrated in gel-filtration buffer. SDS-PAGE confirmed the purity (>98%) of the protein and mass-spectrometry confirmed both the identity of native A52_ΔN36_ and the percentage Se incorporation (100%) of the SeMet-labelled protein (data not shown).

### Purification of B14

Cells were thawed, resuspended in 50 mM Tris pH 8.0, 500 mM NaCl, 1 M non-detergent sulphobetaine 201, 2% v/v Triton X-100 and 2 mM β-mercaptoethanol, cooled on ice and sonicated until the cells were lysed completely. Lysate was cleared by centrifugation (20,000 *g*, 4°C, 30 min) and applied to a 5 mL Ni-Sepharose HiTrap affinity chromatography column (GE Healthcare) that had been equilibrated in binding buffer (30 mM imidazole, 500 mM NaCl, 20 mM Tris pH 7.9, 2 mM β-mercaptoethanol). The column was washed with binding buffer (5 c.v.) and eluted with an increasing gradient of imidazole (30–500 mM). Fractions containing B14 were pooled and applied to a Superdex 75 column (GE Healthcare) equilibrated in 50 mM Tris-HCl pH 8.5, 150 mM NaCl, 2 mM β-mercaptoethanol. SDS-PAGE and mass-spectrometry confirmed the purity (>98%) and identity of the purified protein (data not shown). The presence of β-mercaptoethanol throughout the purification was essential to prevent aggregation and precipitation of the protein.

### Crystallisation and data collection

Purified A52_ΔN36_ and B14 were concentrated to 5.2–5.4 mg/mL and 3.2 mg/mL in 5 kDa MWCO (Vivascience) and 10 kDa MWCO (Millipore) microconcentrators, respectively. Initial screening of crystallisation conditions was performed at 21°C in 96-well plates with sitting drops (100 nL protein+100 nL reservoir) equilibrated against 95 µL of reservoir solution [49]. For unlabelled and SeMet-labelled A52_ΔN36_, initial crystallisation conditions were scaled up as hanging drops (1 µL protein+1 µL reservoir) in 24-well Linbro-style plates equilibrated at 21°C against 500 µL reservoirs containing 15–21% w/v polyethylene glycol 3350 and 0.2–0.24 M tri-sodium citrate (space group *P*2_1_) or 22% w/v polyethylene glycol 3350 and 0.2 M sodium phosphate (space group *R*3), diffraction quality crystals appearing within 48 h. For B14, initial crystallisation conditions were optimized by screening additives [49] and diffraction quality crystals were obtained in 48 h against reservoirs containing 0.2 M di-ammonium tartrate, 20% w/v polyethylene glycol 3350, 0.4 M non-detergent sulphobetaine 201 and 2 mM β-mercaptoethanol.

All crystals were cryoprotected by a quick pass through reservoir solution supplemented with 20% v/v glycerol before being flash-cryocooled in a cold (100 K) stream of N_2_ gas. Diffraction data were collected at 100 K at the ESRF beamlines ID14-4 (high resolution unlabelled A52) and BM14 (SeMet-lebelled B14 and A52) and at Diamond beamline I03 (low resolution unlabelled A52). Diffraction data were processed with HKL2000 [50] (Table 1).

### Structure solution, refinement and validation

The three wavelengths of the A52 MAD experiment were cross-scaled, the selenium sites were located, the selenium substructure was refined, and calculated phases were solvent-flattened with SHELXD [51], SHARP [52], DM [53] and SOLOMON [54] as implemented by the AutoSHARP structure solution pipeline [55]. The chain was traced using ARP/wARP [56], 281 of the 304 residues in the final model being positioned automatically. For all structures, manual model manipulation was performed using COOT [57]. For B14, the three wavelengths of the MAD experiment were cross-scaled and selenium sites were located using SHELXC and SHELXD as implemented in HKL2MAP [58]. Initial phases were calculated using SHARP [59], solvent flattening was performed using RESOLVE [60], and the backbone was traced manually.

Initial structure refinement was performed using CNS [61] (B14) or using REFMAC5 with phase restraints [62] (A52). The high-resolution structure of unlabelled A52 (space group *P*2_1_) was determined directly from the isomorphous structure solved by MAD analysis and the lower-resolution (*R*3) structure by molecular replacement using PHASER [63]. Final refinement was performed using REFMAC5 (high resolution A52) or phenix.refine [64] (B14 and low-resolution A52) in consultation with the validation tools present in COOT and the MolProbity web server [57],[65]. For B14 and the low-resolution structure of A52, non-crystallographic symmetry restraints were used throughout the refinement. Atomic coordinates and structure factors have been deposited with the Protein Data Bank, accession IDs 2vvw (high-resolution A52), 2vvx (low-resolution A52) and 2vvy (B14).

### Structural analysis

The representative set of Bcl-2–like structures used for structure-based phylogenetic analysis were selected with the assistance of the SSM web server [34]. Gap-penalty–weighted pairwise superposition of all Bcl-2–like structures was performed using the program SHP [66] to maximise the sum of probabilities of equivalence between pairs of residues for the proteins being compared [67]. The total summed probability was converted into an estimate of the evolutionary distance using the expression: D = −log[(P-2)/(<N>-2)], where D is the evolutionary distance, P is the sum of probabilities and <N> is the mean number of residues in the two molecules [25]. For crystal structures where more than two Bcl-2–like molecules were present in the asymmetric unit the most representative monomers as determined using the MCentral command of LSQMAN [68] was used for the analysis. For structures with two Bcl-2–like molecules per asymmetric unit ‘chain A’ was chosen arbitrarily. For NMR ensembles, the ‘core’ of the most representative member of the ensemble as determined by the OLDERADO server [69] was used. The tree representation was generated from the matrix of evolutionary distances using the programs FITCH and DRAWTREE, part of the PHYLIP package [70], using default parameters. C^α^ rmsds, numbers of equivalent residues and evolutionary distances used to generate the phylogenetic tree are presented in Table S1.

Structure-based multiple sequence alignments were generated from superposed co-ordinate files (see above) with the “Match->Align” tool in UCSF Chimera [71] using default parameters. Interaction interfaces were analysed using the PISA web server [72].

### Reporter assays

Human embryonic kidney 293 (HEK 293) cells (1×10^5^ per well) were seeded in 24-well plates and transfected with 250 ng of expression plasmid encoding either FLAG-tagged A52, B14, N1, or M11, together with 100 ng of NF-κB-luc reporter plasmid and 10 ng of pTK-*Renilla* luciferase internal control with FugeneHD (Roche) according to the manufacturer's instructions. For analysis of the function of the N terminus of A52, cells were transfected with pOPINE vectors expressing either C-terminally His-tagged full-length A52 or A52_ΔN36_ (described above), together with plasmids encoding the NF-κB reporter and *Renilla* internal control. The total amount of DNA (500 ng) was kept constant by supplementation with pCI (Promega) or pOPINE. After overnight incubation, the transfected cells were simulated with 100 ng/ml of IL-1α or TNFα (Peprotech) for 8 h. Alternatively, cells were transfected with 200 ng of the A52, B14, N1, or M11 expression alleles together with 190 ng TRAF2 or TRAF6, 100 ng of NF-κB-luc reporter plasmid and 10 ng of pTK-*Renilla* luciferase internal control and were incubated for 24 h. Cells were harvested in passive lysis buffer (Promega), and the relative stimulation of NF-κB activity was calculated by normalizing luciferase activity with *Renilla* luciferase intensity. In all cases, data shown are from one of two to four independent experiments with similar qualitative results. Data from experiments performed in triplicate are expressed as means±standard deviation.

### Apoptosis assay

Apoptosis was measured as described previously [26]. Briefly, HeLa cells were transfected with expression vectors for FLAG-tagged A52, B14, N1, M11, untagged Bcl-x_L_ or empty vector (pCI) together with a CD20 surface transfection marker using FugeneHD (Roche). Cells were stimulated with 0.5–1 µM staurosporine for 1 h or left untreated as indicated. The level of apoptosis was assessed by measuring the change in mitochondrial potential (Δψ_m_) using the potentiometric dye JC-1. Cells were collected, washed in phosphate-buffered saline (10 mM phosphate pH 7.4, 137 mM NaCl; PBS) and stained with anti-CD20 APC antibody (BD Pharmingen) for 20 min on ice. Cells were then stained with 2 µM JC-1 dye (Invitrogen) for 30 min at 37 °C, washed in PBS, re-suspended in FACS buffer (PBS with 2% v/v foetal calf serum) and analyzed by flow cytometry (FACScan; Becton Dickinson). Data was analysed using Summit software (Dako). To assess relative protein expression levels, cells remaining after FACS analysis were collected and harvested in RIPA buffer [50 mM Tris-HCl pH 7.4, 150 mM NaCl, 1% Triton v/v X-100, 1% w/v sodium deoxycholate and 0.1% w/v SDS with protease inhibitor cocktail tablets (Roche)]. Lysates were separated by SDS-PAGE (15% gel), transferred to nitrocellulose membranes and blotted for the presence of N1, A52, B14, M11 using anti-FLAG monoclonal antibody (Sigma) or for Bcl-x_L_ using an anti-Bcl-x_L_ monoclonal antibody (Cell Signalling Technologies). Equal protein loading was assessed using an anti-tubulin-α polyclonal antibody (Chemicon).

## Supporting Information

Figure S1 A52 and B14 are dimers. (A) Overlay of the structures of A52 solved in space groups *P*2_1_ (orange) and *R*3 (green). (B) Overlay of the two dimers of B14 present in the asymmetric unit coloured green and magenta, respectively.
(3.51 MB TIF)

Figure S2 Unlike N1, M11 and Bcl-x_L_, A52 and B14 do not inhibit apoptosis. (A) HeLa cells were transfected with vectors expressing FLAG-tagged A52, B14, N1, M11, untagged Bcl-x_L_ or empty control vector (pCI; EV) together with CD20 cell-surface expression vector. After a further 24 h, cells were treated with 1 µM staurosporine for 1 h or left untreated and assayed for mitochondrial dysfunction by JC-1 staining and FACS analysis. Data are expressed as means±standard deviation of 2–4 independent experiments. Statistics: two-tailed Student's t-Test (*P<0.05, **P<0.005, ***P<0.0005). (B) Cells used for FACS analysis in (A) were harvested, normalised for protein content, separated by SDS-PAGE and immunoblotted for N1, B14, A52 and M11 using an anti-FLAG antibody or Bcl-x_L_ using an anti-Bcl-x_L_ antibody. Equal loading is shown by immunoblotting with an anti-tubulin-α antibody.
(0.42 MB TIF)

Figure S3 Multiple sequence alignment of the VACV A52/B14 family of proteins. The alignment shown is based on the Pfam [22] alignment, modified to maximise structural equivalences between A52 and B14 (see Figure 1D). Sequences are from VACV WR for all proteins except C16/B22, which is from VACV Copenhagen, and UniProt accession IDs are shown in parentheses. Residues that are highly or moderately conserved (BLOSUM62 score) are coloured marine and light blue, respectively. The secondary structures of A52 and B14 are shown above the sequences with α helices represented as cylinders. A graph of the conservation score for each residue [73] is shown beneath the sequences.
(1.27 MB TIF)

Table S1 Structural similarity of poxvirus, herpesvirus and cellular Bcl-2–like proteins. The PDB ID of each structure is shown (with chain or member of NMR ensemble used for the analysis shown in parentheses). C^α^ rmsds and number of equivalent residues (in parentheses) are shown above the diagonal, evolutionary distances are shown below.
(0.13 MB DOC)
