# Supplementary figures and images for: Vaccinia Virus Proteins A52 and B14 Share a Bcl-2–Like Fold but Have Evolved to Inhibit NF-κB rather than Apoptosis
Source: PLoS Pathog. 2008 Aug 15;4(8):e1000128. doi: 10.1371/journal.ppat.1000128 (PMC2494871; doi:10.1371/journal.ppat.1000128)

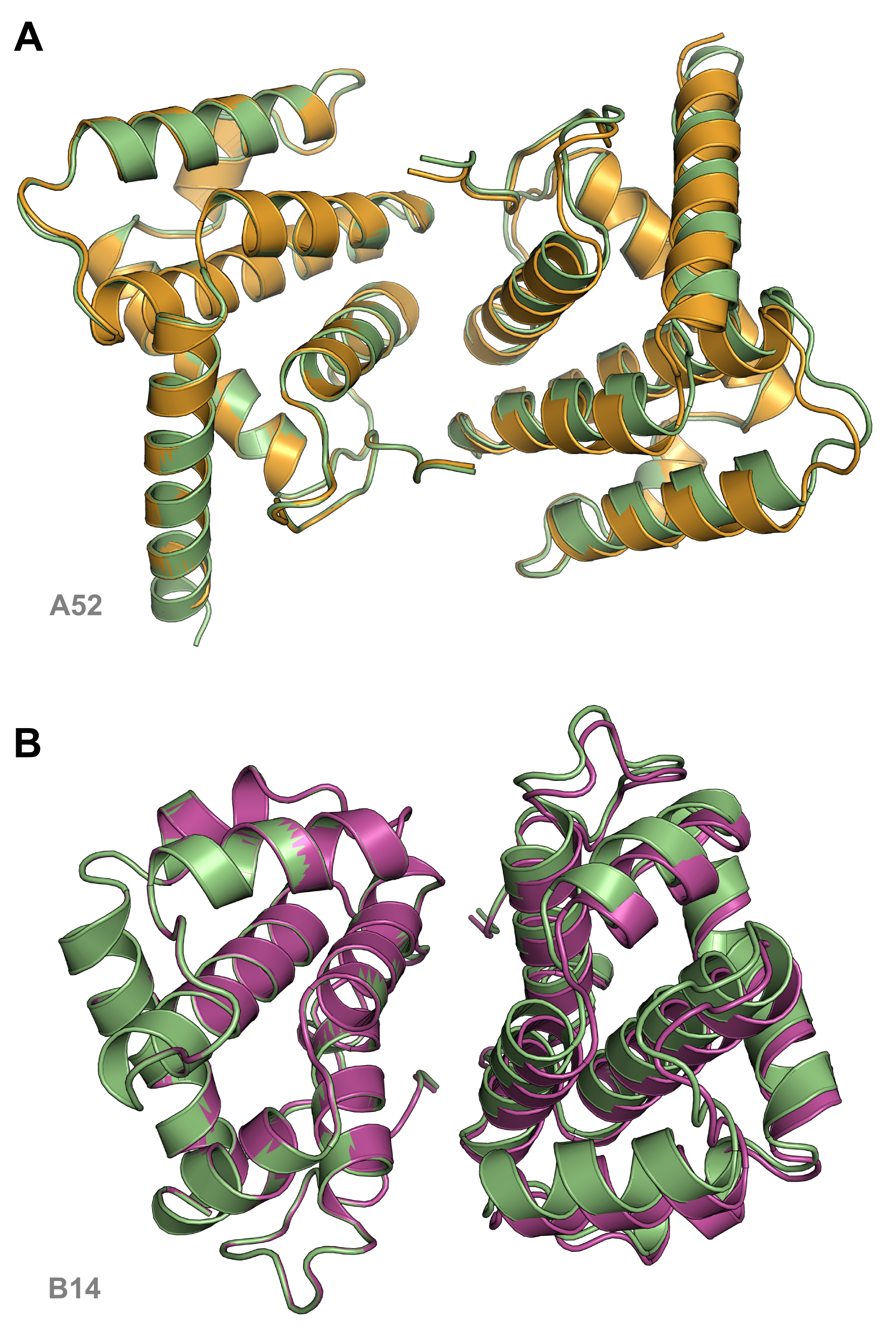

Supplement: Figure S1 — A52 and B14 are dimers. (A) Overlay of the structures of A52 solved in space groups P21 (orange) and R3 (green). (B) Overlay of the two dimers of B14 present in the asymmetric unit coloured green and magenta, respectively. (3.51 MB TIF) [file ppat.1000128.s001.tif]

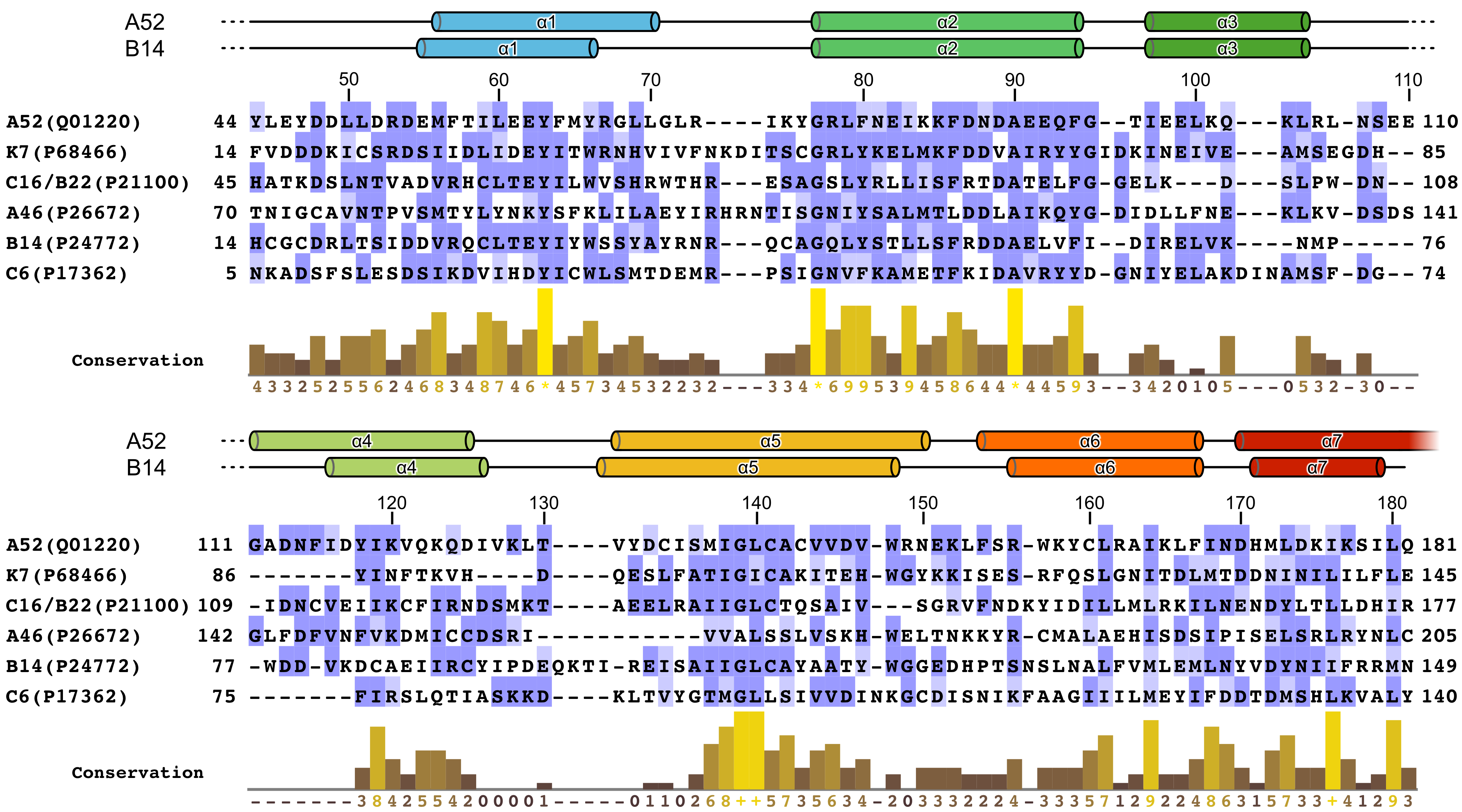

Supplement: Figure S3 — Multiple sequence alignment of the VACV A52/B14 family of proteins. The alignment shown is based on the Pfam [22] alignment, modified to maximise structural equivalences between A52 and B14 (see Figure 1D). Sequences are from VACV WR for all proteins except C16/B22, which is from VACV Copenhagen, and UniProt accession IDs are shown in parentheses. Residues that are highly or moderately conserved (BLOSUM62 score) are coloured marine and light blue, respectively. The secondary structures of A52 and B14 are shown above the sequences with α helices represented as cylinders. A graph of the conservation score for each residue [73] is shown beneath the sequences. (1.27 MB TIF) [file ppat.1000128.s003.tif]
