# Supplementary material for: Vaccinia Virus Proteins A52 and B14 Share a Bcl-2–Like Fold but Have Evolved to Inhibit NF-κB rather than Apoptosis
Source: PLoS Pathog. 2008 Aug 15;4(8):e1000128. doi: 10.1371/journal.ppat.1000128 (PMC2494871; doi:10.1371/journal.ppat.1000128)
Supplement: Table S1 — Structural similarity of poxvirus, herpesvirus and cellular Bcl-2–like proteins. The PDB ID of each structure is shown (with chain or member of NMR ensemble used for the analysis shown in parentheses). Cα rmsds and number of equivalent residues (in parentheses) are shown above the diagonal, evolutionary distances are shown below. (0.13 MB DOC) [file ppat.1000128.s004.doc]

**Table S1.** Structural similarity of poxvirus, herpesvirus and cellular Bcl-2–like proteins. PDB ID of each structure is shown (with chain or member of NMR ensemble used for the analysis shown in parentheses). C^α^ rmsds and number of equivalent residues (in parentheses) are shown above the diagonal, evolutionary distances are shown below.

|  | **1af3(A)** | **1ddb(P)** | **1g5m(A)** | **1k3k(A)** | **1maz(A)** | **1o0l(M)** | **1ohu(A)** | **1pq0(A)** | **1pq1(A)** | **1q59(A)** | **1ty4(A)** | **1wsx(B)** | **2abo(A)** | **2bid(N)** | **2bzw(A)** | **2i39(C)** | **2ims(A)** | **2imt(A)** | **2jbx(A)** | **2jby(A)** | **2jcn(A)** | **2jm6(E)** | **2nl9(A)** | **2nla(A)** | **2pqk(A)** | **2uxe(B)** | **2vm6(A)** | **2vog(A)** | **2voh(A)** | **3bl2(A)** | **2vvw(A)** | **2vvy(A)** |
| --- | --- | --- | --- | --- | --- | --- | --- | --- | --- | --- | --- | --- | --- | --- | --- | --- | --- | --- | --- | --- | --- | --- | --- | --- | --- | --- | --- | --- | --- | --- | --- | --- |
| **1af3(A)** | - | 2.67 (86) | 1.60 (138) | 2.62 (106) | 0.55 (139) | 1.86 (125) | 1.78 (131) | 0.70 (142) | 1.41 (138) | 2.64 (116) | 1.95 (130) | 1.95 (119) | 2.33 (110) | 2.76 (111) | 1.56 (137) | 3.00 (98) | 2.11 (127) | 2.07 (130) | 2.49 (106) | 2.40 (103) | 2.25 (132) | 1.91 (117) | 2.00 (126) | 1.96 (129) | 2.07 (127) | 2.95 (98) | 2.04 (125) | 2.05 (120) | 1.85 (121) | 2.26 (109) | 2.64 (104) | 2.61 (99) |
| **1ddb(P)** | 1.519 | - | 2.78 (81) | 2.77 (66) | 2.72 (83) | 2.87 (75) | 2.80 (80) | 2.54 (84) | 2.79 (79) | 3.06 (82) | 3.08 (79) | 2.29 (78) | 2.79 (85) | 2.51 (84) | 2.94 (49) | 2.76 (63) | 3.03 (81) | 2.98 (76) | 2.87 (71) | 2.90 (39) | 2.93 (82) | 2.59 (77) | 2.46 (75) | 3.09 (75) | 2.69 (78) | 2.84 (64) | 2.74 (75) | 2.72 (74) | 2.83 (78) | 2.77 (36) | 2.76 (73) | 2.91 (70) |
| **1g5m(A)** | 0.958 | 1.700 | - | 2.71 (110) | 1.62 (133) | 1.66 (131) | 2.21 (137) | 1.67 (136) | 1.49 (137) | 2.79 (125) | 2.15 (137) | 1.70 (116) | 2.27 (109) | 2.94 (100) | 1.46 (137) | 2.70 (101) | 2.16 (131) | 2.10 (133) | 2.27 (105) | 2.53 (111) | 2.18 (131) | 2.13 (121) | 2.11 (129) | 2.16 (132) | 2.18 (129) | 2.72 (99) | 2.12 (124) | 2.19 (123) | 2.21 (128) | 2.24 (111) | 2.74 (114) | 2.81 (103) |
| **1k3k(A)** | 1.441 | 1.858 | 1.508 | - | 2.36 (101) | 2.55 (117) | 2.58 (114) | 2.61 (105) | 2.48 (113) | 2.79 (97) | 2.41 (118) | 2.41 (101) | 2.47 (96) | 2.74 (86) | 2.36 (112) | 2.63 (102) | 2.72 (107) | 2.88 (112) | 2.53 (105) | 2.34 (98) | 2.49 (104) | 2.67 (108) | 2.50 (114) | 2.71 (116) | 2.53 (114) | 2.50 (98) | 2.74 (113) | 2.61 (110) | 2.76 (117) | 2.52 (100) | 2.67 (110) | 2.58 (101) |
| **1maz(A)** | 0.751 | 1.498 | 0.991 | 1.450 | - | 1.89 (126) | 1.87 (132) | 0.41 (139) | 1.51 (135) | 2.72 (117) | 1.99 (128) | 1.84 (121) | 2.24 (109) | 2.71 (108) | 1.42 (133) | 2.98 (100) | 2.10 (126) | 2.14 (129) | 2.50 (104) | 2.32 (100) | 2.12 (129) | 2.04 (117) | 2.12 (128) | 1.98 (129) | 2.18 (126) | 2.93 (92) | 2.05 (123) | 1.98 (120) | 1.87 (121) | 2.16 (108) | 2.74 (108) | 2.75 (99) |
| **1o0l(M)** | 1.141 | 1.873 | 1.121 | 1.411 | 1.134 | - | 2.22 (130) | 1.84 (125) | 1.55 (129) | 2.89 (115) | 2.00 (136) | 1.90 (112) | 2.28 (106) | 2.92 (96) | 1.58 (131) | 2.64 (108) | 2.22 (125) | 2.13 (126) | 2.49 (108) | 2.38 (106) | 2.28 (128) | 1.95 (115) | 2.01 (122) | 2.10 (126) | 2.15 (123) | 2.65 (105) | 2.13 (120) | 2.35 (121) | 2.28 (123) | 2.12 (107) | 2.83 (116) | 2.72 (107) |
| **1ohu(A)** | 1.030 | 1.753 | 1.158 | 1.409 | 1.061 | 1.245 | - | 1.99 (133) | 1.85 (128) | 2.79 (127) | 1.23 (151) | 2.29 (123) | 2.45 (99) | 2.67 (98) | 2.01 (130) | 2.68 (99) | 2.41 (125) | 2.34 (125) | 2.44 (107) | 2.35 (105) | 2.57 (128) | 2.16 (116) | 2.29 (122) | 2.08 (124) | 2.30 (122) | 2.66 (97) | 2.33 (117) | 2.38 (117) | 2.38 (119) | 2.48 (109) | 2.84 (105) | 2.57 (103) |
| **1pq0(A)** | 0.732 | 1.514 | 0.978 | 1.441 | 0.740 | 1.135 | 1.046 | - | 1.37 (135) | 2.76 (115) | 2.07 (129) | 1.97 (119) | 2.37 (111) | 2.83 (112) | 1.62 (137) | 2.57 (94) | 2.15 (127) | 2.06 (127) | 2.49 (105) | 2.23 (101) | 2.27 (127) | 2.06 (117) | 2.09 (125) | 1.99 (127) | 2.07 (124) | 2.90 (96) | 2.07 (124) | 2.10 (119) | 1.92 (121) | 2.38 (111) | 2.81 (102) | 2.76 (97) |
| **1pq1(A)** | 0.868 | 1.628 | 0.936 | 1.347 | 0.885 | 1.056 | 1.085 | 0.878 | - | 2.68 (106) | 1.68 (133) | 1.73 (120) | 2.19 (107) | 2.69 (107) | 0.88 (141) | 2.72 (100) | 2.00 (133) | 1.98 (134) | 2.48 (110) | 2.44 (109) | 2.14 (137) | 1.67 (121) | 1.67 (126) | 1.80 (131) | 1.88 (127) | 2.69 (100) | 1.77 (127) | 1.81 (125) | 1.74 (126) | 2.28 (112) | 2.63 (109) | 2.76 (98) |
| **1q59(A)** | 1.429 | 1.818 | 1.448 | 1.685 | 1.413 | 1.607 | 1.420 | 1.449 | 1.489 | - | 2.83 (123) | 2.75 (108) | 2.82 (104) | 3.36 (104) | 2.55 (113) | 2.97 (90) | 2.99 (106) | 3.02 (107) | 2.76 (97) | 2.72 (102) | 3.03 (103) | 2.92 (105) | 2.82 (106) | 2.88 (109) | 2.98 (109) | 2.81 (87) | 2.96 (95) | 2.99 (92) | 3.05 (106) | 2.99 (104) | 2.94 (93) | 2.96 (96) |
| **1ty4(A)** | 1.091 | 1.851 | 1.138 | 1.315 | 1.118 | 1.150 | 0.868 | 1.123 | 1.022 | 1.447 | - | 2.41 (110) | 2.27 (108) | 2.95 (103) | 1.79 (134) | 2.49 (103) | 2.42 (126) | 2.40 (128) | 2.39 (113) | 2.41 (110) | 2.39 (124) | 2.14 (118) | 1.93 (122) | 2.21 (130) | 1.97 (124) | 2.51 (99) | 2.22 (122) | 2.24 (122) | 2.27 (126) | 2.30 (111) | 2.68 (110) | 2.75 (99) |
| **1wsx(B)** | 1.059 | 1.474 | 1.136 | 1.362 | 1.003 | 1.231 | 1.169 | 1.048 | 1.031 | 1.472 | 1.300 | - | 2.09 (105) | 2.82 (97) | 1.76 (119) | 2.51 (96) | 2.01 (117) | 2.03 (119) | 2.17 (102) | 2.42 (106) | 2.16 (123) | 1.43 (121) | 1.75 (127) | 1.42 (125) | 1.65 (123) | 2.59 (96) | 1.94 (117) | 2.06 (119) | 2.00 (120) | 2.23 (105) | 2.51 (106) | 2.69 (101) |
| **2abo(A)** | 1.213 | 1.509 | 1.327 | 1.459 | 1.211 | 1.387 | 1.361 | 1.229 | 1.260 | 1.546 | 1.336 | 1.181 | - | 2.63 (97) | 2.07 (105) | 2.62 (98) | 2.42 (108) | 2.51 (111) | 2.44 (102) | 2.67 (99) | 2.55 (111) | 2.33 (109) | 2.50 (115) | 2.55 (121) | 2.52 (117) | 2.45 (93) | 2.40 (104) | 2.59 (108) | 2.46 (108) | 1.64 (124) | 2.48 (98) | 2.42 (100) |
| **2bid(N)** | 1.395 | 1.458 | 1.644 | 1.693 | 1.404 | 1.718 | 1.591 | 1.407 | 1.414 | 1.792 | 1.532 | 1.479 | 1.464 | - | 2.75 (103) | 2.83 (84) | 3.02 (105) | 2.90 (108) | 2.90 (83) | 3.12 (59) | 3.07 (103) | 2.73 (100) | 2.81 (100) | 2.73 (100) | 2.67 (99) | 3.27 (81) | 2.89 (60) | 2.95 (62) | 2.71 (96) | 2.78 (55) | 2.84 (91) | 2.51 (89) |
| **2bzw(A)** | 0.876 | 1.912 | 0.938 | 1.282 | 0.885 | 1.035 | 1.091 | 0.869 | 0.769 | 1.433 | 1.017 | 1.020 | 1.243 | 1.485 | - | 2.67 (95) | 2.03 (128) | 2.09 (129) | 2.33 (109) | 2.32 (105) | 2.25 (132) | 1.78 (121) | 1.69 (126) | 1.87 (131) | 1.75 (126) | 2.53 (97) | 1.81 (125) | 1.76 (124) | 1.69 (124) | 2.20 (112) | 2.72 (111) | 2.79 (103) |
| **2i39(C)** | 1.525 | 1.769 | 1.496 | 1.365 | 1.511 | 1.404 | 1.461 | 1.462 | 1.411 | 1.729 | 1.363 | 1.317 | 1.344 | 1.618 | 1.396 | - | 2.50 (101) | 2.58 (99) | 2.29 (101) | 2.22 (99) | 2.68 (105) | 2.48 (60) | 2.68 (99) | 2.71 (103) | 2.74 (104) | 0.38 (114) | 2.66 (99) | 2.60 (95) | 2.68 (104) | 2.36 (97) | 1.93 (107) | 2.29 (101) |
| **2ims(A)** | 1.139 | 1.800 | 1.176 | 1.544 | 1.146 | 1.275 | 1.308 | 1.159 | 1.083 | 1.726 | 1.307 | 1.175 | 1.384 | 1.618 | 1.097 | 1.441 | - | 0.15 (161) | 2.39 (107) | 2.53 (111) | 0.98 (153) | 1.95 (120) | 2.17 (125) | 2.18 (130) | 2.23 (128) | 2.64 (103) | 1.99 (124) | 1.81 (124) | 1.87 (127) | 2.34 (114) | 2.97 (112) | 2.85 (102) |
| **2imt(A)** | 1.118 | 1.840 | 1.141 | 1.544 | 1.129 | 1.258 | 1.293 | 1.142 | 1.072 | 1.735 | 1.291 | 1.165 | 1.366 | 1.501 | 1.115 | 1.489 | 0.714 | - | 2.49 (109) | 2.46 (113) | 1.00 (155) | 1.95 (122) | 2.16 (128) | 2.14 (131) | 2.23 (131) | 2.73 (104) | 1.97 (126) | 1.89 (128) | 1.87 (129) | 2.28 (113) | 3.07 (110) | 3.07 (111) |
| **2jbx(A)** | 1.364 | 1.759 | 1.377 | 1.378 | 1.359 | 1.407 | 1.395 | 1.366 | 1.320 | 1.585 | 1.324 | 1.258 | 1.341 | 1.737 | 1.281 | 1.221 | 1.385 | 1.393 | - | 1.14 (121) | 2.53 (113) | 2.30 (103) | 2.53 (113) | 2.55 (111) | 2.44 (114) | 2.29 (101) | 2.73 (106) | 2.80 (106) | 2.71 (109) | 2.38 (105) | 2.46 (107) | 2.46 (95) |
| **2jby(A)** | 1.317 | 1.715 | 1.362 | 1.358 | 1.305 | 1.419 | 1.382 | 1.298 | 1.293 | 1.532 | 1.353 | 1.222 | 1.415 | 1.694 | 1.277 | 1.199 | 1.385 | 1.332 | 0.839 | - | 2.66 (115) | 2.23 (103) | 2.43 (111) | 2.36 (109) | 2.35 (110) | 2.26 (99) | 2.46 (104) | 2.44 (105) | 2.56 (111) | 2.53 (98) | 2.56 (105) | 2.76 (96) |
| **2jcn(A)** | 1.106 | 1.724 | 1.166 | 1.507 | 1.117 | 1.278 | 1.313 | 1.126 | 1.061 | 1.748 | 1.291 | 1.136 | 1.342 | 1.574 | 1.105 | 1.414 | 0.803 | 0.789 | 1.391 | 1.366 | - | 2.05 (120) | 2.22 (128) | 2.15 (132) | 2.21 (130) | 2.68 (98) | 2.14 (131) | 2.03 (128) | 2.16 (132) | 2.25 (109) | 2.78 (115) | 3.03 (106) |
| **2jm6(E)** | 1.082 | 1.575 | 1.131 | 1.401 | 1.083 | 1.233 | 1.227 | 1.124 | 1.014 | 1.573 | 1.185 | 0.903 | 1.198 | 1.452 | 1.026 | 1.344 | 1.134 | 1.117 | 1.276 | 1.238 | 1.138 | - | 1.16 (127) | 0.93 (130) | 1.23 (127) | 2.70 (97) | 1.78 (121) | 1.80 (120) | 1.97 (124) | 2.15 (108) | 2.53 (109) | 2.76 (102) |
| **2nl9(A)** | 1.087 | 1.662 | 1.171 | 1.358 | 1.092 | 1.235 | 1.253 | 1.106 | 1.034 | 1.580 | 1.190 | 0.985 | 1.270 | 1.524 | 1.039 | 1.437 | 1.216 | 1.200 | 1.292 | 1.314 | 1.173 | 0.899 | - | 1.02 (138) | 0.31 (142) | 2.77 (100) | 1.79 (131) | 2.09 (134) | 1.84 (131) | 2.14 (112) | 2.77 (118) | 2.77 (105) |
| **2nla(A)** | 1.060 | 1.889 | 1.120 | 1.391 | 1.063 | 1.217 | 1.188 | 1.080 | 1.001 | 1.618 | 1.168 | 0.932 | 1.233 | 1.486 | 1.010 | 1.388 | 1.141 | 1.126 | 1.357 | 1.265 | 1.135 | 0.835 | 0.850 | - | 1.19 (144) | 2.69 (101) | 1.78 (131) | 2.01 (131) | 1.90 (134) | 2.21 (114) | 2.65 (115) | 2.77 (103) |
| **2pqk(A)** | 1.084 | 1.683 | 1.163 | 1.330 | 1.083 | 1.205 | 1.240 | 1.097 | 1.034 | 1.637 | 1.155 | 0.956 | 1.245 | 1.507 | 1.022 | 1.372 | 1.179 | 1.164 | 1.252 | 1.230 | 1.145 | 0.876 | 0.752 | 0.802 | - | 2.74 (103) | 1.99 (134) | 1.98 (131) | 1.89 (134) | 2.15 (114) | 2.77 (116) | 2.84 (107) |
| **2uxe(B)** | 1.509 | 1.755 | 1.524 | 1.356 | 1.539 | 1.443 | 1.470 | 1.512 | 1.424 | 1.677 | 1.398 | 1.337 | 1.354 | 1.771 | 1.370 | 0.716 | 1.430 | 1.435 | 1.225 | 1.194 | 1.459 | 1.367 | 1.443 | 1.377 | 1.362 | - | 2.87 (101) | 2.69 (97) | 2.79 (103) | 2.49 (98) | 1.94 (105) | 2.30 (99) |
| **2vm6(A)** | 1.054 | 1.718 | 1.148 | 1.398 | 1.050 | 1.251 | 1.270 | 1.071 | 1.005 | 1.669 | 1.208 | 1.072 | 1.309 | 2.128 | 1.022 | 1.404 | 1.123 | 1.107 | 1.411 | 1.311 | 1.120 | 1.025 | 1.008 | 0.988 | 1.004 | 1.432 | - | 1.07 (142) | 0.99 (142) | 2.49 (109) | 2.77 (111) | 2.92 (99) |
| **2vog(A)** | 1.112 | 1.728 | 1.192 | 1.404 | 1.109 | 1.278 | 1.290 | 1.126 | 1.041 | 1.765 | 1.213 | 1.097 | 1.343 | 2.088 | 1.027 | 1.435 | 1.104 | 1.089 | 1.412 | 1.326 | 1.108 | 1.045 | 1.039 | 1.027 | 1.026 | 1.432 | 0.750 | - | 0.86 (140) | 2.52 (105) | 2.71 (110) | 2.85 (101) |
| **2voh(A)** | 1.085 | 1.718 | 1.176 | 1.380 | 1.087 | 1.267 | 1.296 | 1.102 | 1.038 | 1.690 | 1.208 | 1.090 | 1.355 | 1.527 | 1.037 | 1.403 | 1.116 | 1.096 | 1.406 | 1.322 | 1.135 | 1.026 | 1.043 | 1.010 | 0.991 | 1.431 | 0.777 | 0.793 | - | 2.45 (114) | 2.78 (116) | 2.75 (105) |
| **3bl2(A)** | 1.231 | 2.494 | 1.280 | 1.393 | 1.199 | 1.328 | 1.359 | 1.224 | 1.196 | 1.624 | 1.286 | 1.223 | 0.906 | 1.634 | 1.165 | 1.287 | 1.275 | 1.277 | 1.270 | 1.357 | 1.290 | 1.175 | 1.198 | 1.203 | 1.172 | 1.320 | 1.311 | 1.333 | 1.273 | - | 2.49 (100) | 2.55 (105) |
| **2vvw(A)** | 1.485 | 1.779 | 1.501 | 1.454 | 1.488 | 1.553 | 1.616 | 1.532 | 1.462 | 1.802 | 1.520 | 1.381 | 1.482 | 1.691 | 1.433 | 1.158 | 1.594 | 1.600 | 1.349 | 1.397 | 1.485 | 1.364 | 1.427 | 1.400 | 1.427 | 1.158 | 1.482 | 1.462 | 1.445 | 1.423 | - | 1.91 (124) |
| **2vvy(A)** | 1.517 | 1.804 | 1.584 | 1.483 | 1.539 | 1.511 | 1.505 | 1.533 | 1.535 | 1.731 | 1.592 | 1.443 | 1.398 | 1.539 | 1.479 | 1.258 | 1.597 | 1.596 | 1.444 | 1.489 | 1.592 | 1.415 | 1.483 | 1.536 | 1.465 | 1.271 | 1.595 | 1.568 | 1.477 | 1.350 | 1.091 | - |
